# Multidisciplinary Approach to the Diagnosis and In-Hospital Management of COVID-19 Infection: A Narrative Review

**DOI:** 10.3389/fphar.2020.572168

**Published:** 2020-12-09

**Authors:** Giuliano Lo Bianco, Santi Di Pietro, Emilia Mazzuca, Aurelio Imburgia, Luca Tarantino, Giuseppe Accurso, Vincenzo Benenati, Federica Vernuccio, Claudio Bucolo, Salvatore Salomone, Marianna Riolo

**Affiliations:** ^1^ Department of Biomedical and Biotechnological Sciences, School of Medicine, University of Catania, Catania, Italy; ^2^ Anesthesiology and Pain Department, Fondazione Istituto G.Giglio, Cefalù, Italy; ^3^ Emergency Medicine Fellowship Programme, University of Pavia, Pavia, Italy; ^4^ Emergency Department, St Mary's Hospital, Imperial College Healthcare NHS Trust, London, United Kingdom; ^5^ Unità operativa Complessa di Pneumologia, A.O. Ospedali Riuniti Villa Sofia Cervello, Palermo, Italy; ^6^ San Marino State Hospital, Cailungo, San Marino Republic; ^7^ Cliniche Humanitas Gavazzeni, U.O. Elettrofisiologia, Bergamo, Italy; ^8^ Department of Surgical, Oncological and Oral Science (Di.Chir.On.S.), Section of Anaesthesia, Analgesia, Intensive Care and Emergency, Policlinico Paolo Giaccone, University of Palermo, Palermo, Italy; ^9^ Polyclinique du Maine, Laval, Pays de la Loire, France; ^10^ Section of Radiology, Department of Biomedicine, Neurosciences and Advanced Diagnostics (BIND), University of Palermo, Palermo, Italy; ^11^ Struttura Complessa di Neurologia, Ospedale Santa Croce di Moncalieri, Asl TO5, Moncalieri (TO), Italy

**Keywords:** COVID-19, hospital care, multispecialist care, pandemic (COVID-19), hospital response capability

## Abstract

Severe Acute Respiratory Syndrome Coronavirus 2 (SARS-CoV-2 or COVID-19 disease) was declared a pandemic on 11th March 2020 by the World Health Organization. This unprecedented circumstance has challenged hospitals’ response capacity, requiring significant structural and organizational changes to cope with the surge in healthcare demand and to minimize in-hospital risk of transmission. As our knowledge advances, we now understand that COVID-19 is a multi-systemic disease rather than a mere respiratory tract infection, therefore requiring holistic care and expertise from various medical specialties. In fact, the clinical spectrum of presentation ranges from respiratory complaints to gastrointestinal, cardiac or neurological symptoms. In addition, COVID-19 pandemic has created a global burden of mental illness that affects the general population as well as healthcare practitioners. The aim of this manuscript is to provide a comprehensive and multidisciplinary insight into the complexity of this disease, reviewing current scientific evidence on COVID-19 management and treatment across several medical specialties involved in the in-hospital care of these patients.

## Introduction

Since COVID-19 has been declared a Public Health Emergency of International concern on the 30th January 2020, more than 39 millions of people worldwide have been infected (https://covid19.who.int/).

Hospitalized patients with COVID-19 require a multidisciplinary approach as the infection can lead to a plethora of clinical scenarios. Most commonly, patients are hospitalized for respiratory insufficiency, which requires oxygen administration delivered in several forms including mechanical ventilation in the most severe cases (Marini and Gattinoni, 2020). Indeed, a subgroup of infected individuals—i.e., approximately 5% of COVID-19 patients - rapidly progress to acute respiratory distress syndrome (ARDS), often associated with multiple organ failure (MOF), sepsis and septic shock requiring admission to intensive care units (ICUs) (Marini and Gattinoni, 2020).

Central nervous system involvement in COVID-19 infection has been noticed since the early stages of the pandemic. In fact, symptoms such as anosmia or ageusia are relatively common among infected individuals (Guan et al., 2020). In addition, other neurological manifestations have been reported, such as meningoencephalitis and stroke, the latter being related to the prothrombotic state observed with this infection (Zhang et al., 2020a). Gastrointestinal symptoms (GIS), as nausea, vomiting, abdominal pain and diarrhea, may be an early manifestation of COVID-19 disease, and some studies suggest that the presence of GIS may indicate a higher probability of a severe course (Jin et al., 2020b; Guan et al., 2020).

Cardiovascular manifestations of COVID-19 such as myocarditis, arrhythmias, acute coronary syndromes and venous thromboembolism also dictate hospital admission and ad-hoc treatment (Clerkin et al., 2020). Eventually, a global burden of mental illness has been associated with the current pandemic. Indeed, these unprecedented circumstances have induced adverse psychological outcomes in the general population as well as among healthcare workers, which range from anxiety, depression or fear up to violence and suicidal ideation (Kisely et al., 2020; Rossi et al., 2020).

This paper reviews the available literature, guidelines and guidance models from multiple medical societies until 3rd October 2020. It aims to provide multidisciplinary guidance for hospital clinicians who are currently involved in the management of COVID-19 patients.

### Management of COVID-19 Patients in the Emergency Department

#### Structural, Organizational and Logistical Response to the Pandemic

Emergency Departments (EDs) are playing on a global scale a pivotal role in providing an adequate response to healthcare demand during COVID-19 pandemic. Traditionally, EDs have to both guarantee emergency care to patients hospitalized via pre-hospital emergency services, and triage and treat a vast number of self-presenting patients on a 24/7 basis. In current pandemic times many EDs are experiencing an unprecedented surge of caseload, that can easily overwhelm ED’s response capacity, already chronically affected by understaffing, overcrowding, limited resources and poor infrastructures. Moreover, ED’s overcrowding during a pandemic can dramatically increase the risk of facilitating and catalyzing the spread of infection among patients and operators. Therefore, now more than ever efforts should be made at all levels to minimize inappropriate ED utilization (Karan, 2020) and to adequately triage patients suspected of SARS-CoV-2 identifying those requiring hospitalization.

The increased demand in health care and the need to guarantee workers and patients safety during epidemic has led to the need of structural, organizational and logistical changes of EDs. Resilience capacity and preparedness of EDs to adapt to the pandemic might be facilitated by the pre-existence of institutional mass casualties' incidents (MCI) protocols. However, several differences exist between MCI and a viral pandemic. Indeed, MCI typically have a finite number of cases concentrated in an initial peak followed by a progressive decline of visits and hospital admissions over time. In addition to that, managing patients during MCI usually does not require the rigid need of standard precautions as during COVID-19 pandemic. Nevertheless, when facing a viral outbreak, prediction models can help hospitals to adapt their response and the deployment of resources (Gagliano et al., 2020; Paganini et al., 2020).

Interestingly, during the ongoing pandemic similar solutions have been adopted by authors working in very different contexts across the globe. Tents or tent-like structures have been widely used in several countries. Typically positioned outside EDs, these structures offer a simple solution to expand ED’s spaces and can serve as waiting areas as well as for the initial triage of patients (Chen et al., 2020a; Liang et al., 2020; Paganini et al., 2020).

In currently available literature, there is wide agreement on the necessity of creating two distinct and physically separated pathways for suspected COVID-19 patients (also defined as “dirty” or “red” or “infectious” pathway) from non-COVID-19 patients (often referred as “green” or “clean” pathway) (Chen et al., 2020a; Asperges et al., 2020; Gagliano et al., 2020; Liang et al., 2020; Paganini et al., 2020; Paglia et al., 2020). This can be achieved by readapting other hospital buildings in the hospital or alternatively by expanding ED’s space into adjacent repurposed areas. Another possibility is represented by the creation of a filter zone inside ED’s pre-existing spaces by using available construction plastic, similar to the barriers in the refrigeration compartment of stores that are suspended to guarantee a droplet barrier (Paganini et al., 2020).

Whatever areas are repurposed to serve as temporary COVID-19 EDs, it is essential to share decisions with all hospital stakeholders and particularly with hospital engineers: in fact, technical interventions may be needed to ensure availability of oxygen and compressed air, and correct functioning of vacuum and electrical circuits (Paganini et al., 2020). Each area of the ED, i.e., the red and green areas, should use dedicated equipment (e.g., ECG, ultrasound and x-ray machines etc.), to avoid risk of contact-related contagion. Moreover, using portable imaging devices will reduce the need to transfer patients through the hospital, thus helping to reduce droplet spreading (Asperges et al., 2020; Gagliano et al., 2020).

Eventually, during the course of the current COVID-19 epidemic some institutions have implemented strategies of technology-based clinical evaluation (Turer et al., 2020; Wittbold et al., 2020). The adoption of such methods of digital care delivery in Emergency departments is showing promising results, as they can help minimizing direct contact between operators and infectious patients, hence increasing operator’s safety, at the same time also reducing the utilization of personal protective equipment.

#### Triage

One of the greatest difficulties encountered by ED’s healthcare professionals during the current pandemic is certainly represented by triage. Indeed, COVID-19 patients can present with respiratory syndromes indistinguishable from other common conditions. Moreover, respiratory symptoms may even be absent, with patients complaining only of fever and/or a number of other systemic symptoms (Lu et al., 2020a). The variegated clinical picture poses a challenge for early detection during triage at the emergency department (ED). Initially, the World Health Organization (WHO) triage recommendation focused on patients with pneumonia and a recent travel history to Wuhan, based on the knowledge of the outbreak at that time (Global Surveillance, 2020). These criteria were broadened from the 27th of February 2020, to include all patients with acute respiratory disease with no alternative etiology and a history of residence in any country reporting current outbreaks (Liang et al., 2020). Interestingly, a recent study from Singapore demonstrated that using broader ED’s triage criteria as compared to official recommendations, can increase the sensitivity of detection of COVID-19 cases (Liang et al., 2020). Similarly, many healthcare trusts, hospitals and scientific medical societies worldwide have proposed a various number of different triage criteria (Paglia et al., 2020; RCEM Quality Policy, 2020). A higher sensitivity of screening of COVID-19 patient’s at triage will automatically translate into increased inpatient resource utilization, in particular a higher requirement of hospital beds. However, such effort allows to guarantee adequate separation of patients into the most appropriate pathway and reduces the risk of nosocomial transmission of the virus (Liang et al., 2020). In any case, there will always be a risk of nosocomial transmission from asymptomatic COVID-19 patients admitted for other reasons. Hence, each patient accessing the hospital should be considered infectious until proven otherwise and should therefore be provided with a surgical mask (Paglia et al., 2020).

#### Laboratory Test

A baseline number of laboratory investigations, including full blood count, serum electrolytes, renal and hepatic function and coagulation study should be performed in all suspected COVID-19 cases (Paglia et al., 2020; RCEM Quality Policy, 2020). Patients usually show normal leukocytes count and lymphopenia even though leukopenia and leukocytosis have been reported (Rodriguez-Morales et al., 2020). C-reactive protein, hsTroponin, D-Dimer, serum ferritin and lactate dehydrogenase have shown to have prognostic value in initial studies (Tan et al., 2020; Zhou et al., 2020). Blood cultures and testing for atypical bacteria should also be considered (Turer et al., 2020; Rodriguez-Morales et al., 2020).

Procalcitonin is known to be a useful marker to guide initiation and duration of antibiotic treatment in respiratory infections, and preliminary experience in COVID-19 patients seems to confirm current knowledge (Zhou et al., 2020; Schuetz et al., 2017). In particular, dosage of procalcitonin can help in guiding antibiotic appropriateness and in reducing duration of treatments and antibiotic-related side effects (Schuetz et al., 2017). Pulse oximetry should be performed both at rest and after exercise (i.e., 6-min walking test), because a measurement of oxygen saturation at rest only may not detect an underlying respiratory insufficiency (Paglia et al., 2020; RCEM Quality Policy, 2020; FADOI, 2020 ). A more accurate and reliable assessment of the respiratory status and oxygen requirement can be easily obtained with an arterial blood gas analysis, which is recommended as a baseline test by several scientific societies and institutions (Paglia et al., 2020; RCEM Quality Policy, 2020; FADOI, 2020 ). Similarly, nasopharyngeal swabs should be routinely performed in all patients investigated for possible COVID-19 disease, as suggested by WHO guidelines (Paglia et al., 2020; RCEM Quality Policy, 2020; FADOI, 2020 ). SARS-CoV-2 can be detected 1–2 days before the onset of symptoms in upper respiratory tract samples and usually persist for 7–14 days, although cases of prolonged swab positivity have been reported (World Health Organization, 2020c).

Standard precautions assume that every person is potentially infected or colonized with a pathogen that could be transmitted in the healthcare setting.

The swab is also an effective tool for contact-tracing and it is useful to implement prevention and control measures.

In relation to each nation'|’s testing ability, European Center for disease Prevention and Control recommends nasopharyngeal swab in the following categories (presented in order of importance) (European Center for Disease Prevention and Control (ECDC), 2020a; European Center for Disease Prevention and Control (ECDC), 2020b; European Center for Disease Prevention and Control (ECDC), 2020c):

- Patients hospitalized with severe acute respiratory infection for a better clinical management and to provide the rapid patient isolation and implementation of individual protection measures.
- All cases of acute respiratory infection in hospitalized patients or long-term care facilities in order to draw up a prevention program for dedicated staff and for the early treatment of fragile patients.
- All patients admitted to sentinel hospitals with severe acute respiratory infection in order to assess virus circulation in the population.
- Elderly patients and patients with multiple comorbidities to prevent any worsening of the respiratory picture.

#### Imaging

Baseline chest radiographs have a sensitivity for the diagnosis of COVID-19 of 69% (Ambrose et al., 2019). As such, chest radiographs are of little diagnostic value in early stages and its routine use as a screening tool in the early course of the disease is not recommended, except in very exceptional resource-constrained environments (Salehi et al., 2019; Rubin et al., 2020). The main role of chest radiographs is played for assessing disease progression in hospitalized patients, bacterial superinfection, pneumothorax and pleural effusion (Salehi et al., 2019).

Chest CT has a very high sensitivity (97%) for the diagnosis of COVID-19 disease, but a low specificity (i.e., 25–56%), due to overlapping of imaging features of other viral or atypical pneumonia or with non-infectious diseases, such as vasculitis, dermatomyositis (Jin et al., 2020a; Caruso et al., 2020; Kooraki et al., 2020; Tao et al., 2020).

The main findings of COVID-19 patients on both chest radiographs and CT include bilateral pneumonia in the majority of hospitalized patients, with the most common pattern being ground-glass opacities (GGO) with peripheral distribution and predominant involvement of the lower lung zones (Ambrose et al., 2019; Rodriguez-Morales et al., 2020). CT findings of COVID-19 pneumonia vary with time (Bernheim et al., 2020), from single or multiple focal GGO in the early stage, followed by multiple scattered patchy or agglomerated ground-glass opacities that may progress to multiple patchy consolidations (Jin et al., 2020a). In addition, CT angiography can play a role in identifying pulmonary embolism, whose occurrence seems to be higher in COVID-19 patients and it should be suspected especially with evidence of high D-dimer levels (Helms et al., 2020). However, it may be logistically difficult to follow up hospitalized patients with multiple CT scans.

Although there is limited experience at this time on lung ultrasound (LUS) in COVID-19 patients, abundant literature supports the utility of lung ultrasound for a variety of respiratory conditions, including ARDS (Chiumello et al., 2018; Mojoli et al., 2019). This imaging technique offers some advantages over CT: it can be used in the ED or in the prehospital setting for a rapid triage of suspected cases (rule-in/rule-out) and therefore aid decision making for “red” or “green” pathway; it can help to quantify the severity of the disease, thus allowing for prognostic stratification; it can be repeated on patients admitted to hospital to monitor the progression of the disease and efficacy of therapeutic measures (Soldati et al., 2020a); it can be used to diagnose or rule out pneumothorax at the bedside, which is a potential complication of non-invasive and invasive ventilation (Carron, 2020). The main LUS findings in COVID-19 patients are thickening and irregularities of the pleural line, B lines in a variety of patterns including (focal, multifocal, and confluent), consolidations and pleural effusions (the latter two mainly observed in case of superimposed bacterial pneumonia) (Peng et al., 2020).

Nevertheless, lung ultrasound should be performed by experienced physicians whose competencies have been objectively evaluated, and technique and reporting should be standardized as much as possible to facilitate reproducibility between physicians (Soldati et al., 2020b; Di Pietro et al., 2020).

All the above investigations, together with a thorough physical examination and history, will help the emergency physician to stratify the severity of COVID-19 patients and will aid decision making on admission and discharge. Based on current evidence and recommendations, patients should be discharged only when showing no signs of respiratory insufficiency and no requirement of oxygen, i.e., when normal arterial blood gas and saturation both at rest and after physical effort can be demonstrated. Beside the latter investigations, physicians should attentively observe and report the mechanics and work of breathing (Paglia et al., 2020; RCEM Quality Policy, 2020; FADOI, 2020).

#### Treatment and Palliative Care

Available treatments for the management of COVID-19 cases, including modalities of oxygen administration and ventilation as well as pharmacological interventions, will be discussed further below in this review.

In addition to therapeutic interventions, EDs and other wards involved in the care of COVID-19 cases should set up high-quality palliative care pathways to ensure adequate and compassionate end of life care. This should ideally be accomplished through a multidisciplinary cooperation involving experts from relevant specialties (Fausto et al., 2020; Hendin et al., 2020).

#### In-Hospital Infection Control

As a suspected or confirmed COVID-19 patient enters the hospital, prevention of infection spread must be assured. In this regard, the use of Personal Protective Equipment (PPE) is essential for healthcare personnel (HCP), together with general hygiene rules (such as emphasized hand hygiene) (Interim Infection Prevention, 2020).

Ideally, suspected cases should be isolated as soon as possible in separated and well-ventilated areas, preferably a private room with door closed and a private bathroom. Airborne Infection Isolation Rooms (AIIRs) should be used for aerosol generating procedures, however their availability is limited in many hospitals (Saravia et al., 2007). Other interventions, such as cancellation of elective surgical procedures and the implementation of telemedicine-based strategies can help to diminish the number of people accessing the hospital (https://www.cms.gov/files/document/cms-non-emergent-elective-medical-recommendations.pdf; https://www.ama-assn.org/system/files/2020-05/state-elective-procedure-chart.pdf).

### A Multiorgan Disease: Pulmonary Involvement of COVID-19 Infection

COVID-19 is characterized in the majority of cases by a mild respiratory disease, while in approximately 15% of cases a severe pneumonia is observed. The latter can progress to bilateral multifocal pneumonia, leading in 5% of total cases to ARDS, sepsis and septic shock (Wu and McGoogan, 2020).

During the incubation and in non-severe stages, a specific adaptive immune response is activated to eliminate the virus, but the development of this response can be possible if the host is healthy and with an appropriate genetic background (e.g., HLA). Conversely, when immune response is impaired, the virus will propagate and massive destruction of the affected tissues will occur, especially in organs that have high ACE2 expression (Shi et al., 2020). The damaged cells induce innate inflammation in the lungs that is largely mediated by pro-inflammatory macrophages and granulocytes. Lung inflammation is the main cause of life-threatening respiratory disorders at a severe stage (Xu et al., 2020b).

As such, we can distinguish different stages of disease progression with different clinical syndromes:

*Early infection phase* the initial inflammatory response may cause in about 85% of cases mild illness with local or non-specific systemic symptoms such as fever (88–99% of cases), fatigue (38–70%), dry cough (59–68%), anorexia (40%), myalgias (15–35%), dyspnea (19–31%), sputum production (27–34%). Gastrointestinal symptoms (nausea, diarrhea), rhinorrhea, sore throat and pharyngalgia have also been reported (Lechien et al., 2020; Tinku, 2020). These patients usually show no hypoxia on blood gas analysis (BGA), present with a respiratory rate (RR) less than 22 breaths/minute (b/m) and a negative chest radiograph. Most of them do not progress beyond this phase and their management should be assigned to general practitioners (GP) (Lechien et al., 2020; Lopes et al., 2020; Tinku, 2020).

*Pulmonary phase* SARS-CoV-2 shows on its surface a glycoprotein that binds angiotensin-converting enzyme 2 (ACE2), a receptor located on type 2 pneumocytes. Through this way the virus infiltrates the lung parenchyma and begins to proliferate (Li and Ma, 2020). Increased levels of ACE2 were found in SARS-CoV-2 infected cells, suggesting that ACE2 is also involved in post-infection regulation, including immune response, cytokine secretion, and viral genome replication (Li and Ma, 2020). About 15% of infected individuals develop a severe pneumonia with ARF requiring hospitalization and oxygen support. This group of patients need to be closely monitored as some of them may further exacerbate and develop a severe hyperinflammatory response (Tinku, 2020).

*Hyperinflammatory phase* in patients with severe clinical manifestation of COVID-19 a cytokine storm syndrome (CSS) may occur. The hallmark of CSS is an uncontrolled activation and amplification of the host immune system induced by SARS-Cov-2 infection, causing a systemic massive release of proinflammatory cytokines such as TNF-a, IL-1, IL-6 due to the lysis of cells (Heimfarth et al., 2020).

COVID-19 patients with severe symptoms exhibit an extreme decline in total CD4^+^ and CD8^+^ T cells in their circulation: IL-6 may induce apoptosis of T cells through the Fas/FaL pathway, while TNF-a and IFN-I may promote the attachment and retention of T cells in lymphoid organs (Fouladseresht et al., 2020).

CSS could also cause an increase in vascular permeability, resulting in severe damage of the alveolar cells and consequently development of acute respiratory failure (Leiva-Juárez et al., 2018; Zhang et al., 2020b).

Acute respiratory distress syndrome (ARDS) can be observed in these patients, which is characterized by several features, among which a P/F ratio <200 on BGA, increasing of RR above 30 b/m and bilateral opacities at imaging (Vernuccio et al., 2020). An in depth discussion of ARDS will be presented later in this review.

*Bronchoscopic procedures* SarS-CoV-2 can be detected on 93% of bronchoalveolar lavage samples, thus showing a high sensitivity (Wang et al., 2020b). However, its routinary use has been discouraged due to the high risk of contagion to healthcare professionals (Wang et al., 2020b). Nevertheless, bronchoscopy should be considered in specific circumstances such as massive hemoptysis, acute foreign body aspiration, severe central airway obstruction, neutropenic fever with infiltrates and no clinical diagnosis or improvement (Pritchett et al., 2020).

*Oxygen and Ventilatory therapy* According to the ITS- AIPO- SIC document (Harari et al., 2004) patients should be divided into four groups according to their respiratory status:

1. green: SaO 2 > 94%, RR < 20 b/m: if no ARF on BGA, no oxygen needed;
2. yellow: SaO2 <94%, RR > 20 b/m: oxygen supply (up to 10–15 L/min) improves saturation;
3. orange: SaO2 <94%, RR > 20 b/m: poor response to oxygen 10–15 L/min and needing high flow nasal oxygen (HFNO), continuous positive airway pressure (CPAP), NIV with very high FiO2;
4. red: SaO2<94%, RR > 20 b/m: no response to all previous treatments or presenting respiratory distress with PaO2/FiO2 <200 and needing endotracheal intubation (EI).

O2 saturation and RR should be re-evaluated no more than 2 h after therapy initiation and subsequently every 6 h (if target saturation and RR values are met and the patient remains stable) (Harari et al., 2004). High flow nasal cannula (HFNO) may be used as a bridge between oxygen and CPAP (continuous positive airway pressure) trial although this technique generates a relatively high amount of droplets (Harari et al., 2004). Ideally, CPAP should be delivered via a full-face non-vented mask, together with an expiratory viral filter and exhalation port; alternatively, an helmet can be used (as second choice) (Harari et al., 2004). Recommended values for positive end expiratory pressure (PEEP) are between 10 and 15 cmH2O (Harari et al., 2004; NHS Specialty Guides, 2020).

NIV should be used with a full-face non-vented mask and double circuit. Suggested initial settings are PS 8–10 cmH2 0 + 60–100% FiO2. NIV should be considered for hypercapnic respiratory failure or to prevent hypercapnia in COPD patients (NHS Specialty Guides, 2020). Ideally, this should be delivered with a full face non-vented mask and a double circuit, using values of pressure support between 8 and 10 cmH20 (Harari et al., 2004; NHS Specialty Guides, 2020).

In order to improve patient’s comfort and compliance, administration of low doses of opioids can be considered. Humidification is generally discouraged, as it increases the quantity of droplet generation (NHS Specialty Guides, 2020).

Early intubation is mandatory if the patient does not respond adequately to CPAP or NIV (hypoxemia with P/F < 150–175 after 1 h of CPAP/NIV in absence of BGA improvement, RR > 30 b/m; SAPS score >34, intolerance to ventilation, clinical decline) (NHS Specialty Guides, 2020; Antonelli et al., 2001).

Preliminary experience with self-proning of awake non-intubated patients has shown promising results in terms of improving oxygenation levels, although these findings and the safety of the procedure need to be confirmed in further trials (Caputo et al., 2020).

#### Pharmacological Treatment

Numerous studies have been conducted to find potential curative agents against COVID-19 disease, and many trials are still ongoing. Researcher’s attention has been mostly directed towards drugs with direct antiviral activity and to those with immune-modulating or immune-suppressive effects.

Among the antiviral agents, Remdesivir (200 mg loading dose on day 1, followed by 100 mg daily for up to nine additional days) has been demonstrated to be superior to placebo in shortening the time to recovery in adults hospitalized with COVID-19 and evidence of lower respiratory tract infection (Beigel et al., 2020).

A large United Kingdom multicentric study has investigated the role of dexamethasone in hospitalized COVID-19 patients. Investigators have demonstrated a reduction in the 28 days mortality in the intervention group that received 6 mg of dexamethasone (oral or intravenous) for up to 10 days (Horby and Lim, 2020). Interestingly, a subgroup analysis showed that the effects are more pronounced in patients mechanically ventilated or with high oxygen requirements as compared to those with no oxygen requirement (Horby and Lim, 2020). These findings suggest that dexamethasone plays an important role in the modulation of the excessive immune response observed in some cases of COVID-19 (see above *Hyperinflammatory phase*).

The efficacy of several other drugs have been investigated, such as tocilizumab, azithromycin, hydroxychloroquine, however results have been inconclusive (Oldenburg and Doan, 2020; Sanders, 2020; Skipper et al., 2020).

Numerous randomized controlled trials are currently being conducted to assess the efficacy of convalescent plasma (Li et al., 2020a). Current evidence suggests the safety of this therapeutic strategy and has shown promising results, therefore its use has been approved by the FDA and in several european countries (Li et al., 2020b; Shen, 2020).

### A Multiorgan Disease: Gastroenterologic Involvement in COVID-19

Gastrointestinal symptoms (GIS), as nausea, vomiting, abdominal pain and diarrhea, may be an early manifestation of SARS-CoV-2 infection (Wang et al., 2020a; Jin et al., 2020b; Huang et al., 2020). In fact, Huang et al. (2020) reported that GI involvement was present in 2–10% of patients with COVID-19. A systematic review evaluating GI involvement reported that the presence of GI symptoms had a great variability between 2 and 100%; in particular, according to a pooled analysis, 16.1% presented GIS, 8.3% diarrhea, 12% nausea-vomiting and 4% abdominal pain (Pamolona et al., 2020). Sometimes, GI symptoms may precede respiratory ones (Wang et al., 2020a). Some studies suggest that the presence of GIS may indicate a higher probability of a severe course (Jin et al., 2020b; Guan et al., 2020). A higher percentage of diarrhea was observed in patients with severe disease (5.8%) as compared to patients with a mild course of the disease (3.5%) (Guan et al., 2020). As for other organs, also in the GI system the ACE2 receptor plays a fundamental role. This protein, in fact, is expressed in gastric, intestinal and colonic cells, promoting virus infection (Wan et al., 2020a). Therefore, once the virus infects the human intestinal epithelia, it can potentially propagate via fecal-oral route (Wang et al., 2020a; Guan et al., 2020; Pamolona et al., 2020). Interesting, viral RNA is detected in the stool for a longer time than in the respiratory system (Pan et al., 2020; Wu et al., 2020b). Consequently, it has been suggested that its detection in fecal samples should be considered as one of the routine diagnostic tests to guide decision making on hospital discharge and the lifting of isolation measures (Pamolona et al., 2020).

### A Multiorgan Disease: Cardiac and Cardiovascular Involvement of COVID-19 Infection

The myocardial tissue and the cardiovascular (CV) system can be affected by COVID-19 infection through a variety of mechanisms with an important role played by inflammatory cytokines (ESC Guidance, 2020). The main CV manifestations observed are myocarditis, cardiomyopathies, arrhythmias, acute coronary syndromes (STEMI and NSTEMI) and venous thromboembolism which can lead to acute heart failure with cardiogenic shock (Clerkin et al., 2020). Their occurrence is associated with an increased risk of inhospital mortality, so it is crucial to identify these patients as soon as possible. In COVID-19 infection, the severe hypoxia with subsequent increase of circulating catecholamines and the activation of T cells with an abnormal cytokines release (mainly IL-6 and IL-17) lead to oxidative stress and endothelial dysfunction with a consequent microangiopathy, vasospasm and myocardial ischaemia even in absence of coronary lesion. In addition, activation of the immune system leads to plaque instability in coronary arteries leading to coronary lesions, acute myocardial injury and arrhythmias as a consequence (Xu et al., 2020b; Madjid et al., 2020).

It has been reported a high prevalence of CV comorbidities (hypertension, atrial fibrillation (AF), DM, chronic heart failure (CHF) and kidney failure) in COVID patients. In a retrospective analysis carried out on 138 COVID-19 patients in Wuhan, one or more CV comorbidities were found in 50% of cases at least, rising 72% in severe cases (Zhou et al., 2020), with hypertension playing the main role (Wang et al., 2020a; Liu et al., 2020a; Zhou et al., 2020). This detail is important considering that ACE-2 receptor (located also in lungs, heart and vessels) is a part of the renin angiotensin system (RAS) and plays a main role in the development of COVID-19 CV involvement. SARS-CoV-2 infection appears to cause a loss of regulation of the RAS system, leading to upregulation of ACE-2 (Li, 2018; Walls et al., 2020; Zhou et al., 2020). This hypothesis might explain the datum of high prevalence of pre-existing hypertension in COVID-19 patients in ACEinhibitor (ACE-I) or angiotensin receptor blocker (ARBS) treatment, whose cardiac and vascular cells show a major expression of ACE-2 receptors compared to patients who do not assume these drugs (Walls et al., 2020; Zhou et al., 2020). In addition, ACE-2 up-regulation can also cause a direct myocardial injury secondary to an increased catecholamine level (Walls et al., 2020; Zhou et al., 2020).

As expected, common symptoms of CV involvement are represented by chest pain, breathlessness, tachycardia (ESC Guidance, 2020) and other varying signs and symptoms depending on the particular CV manifestation.

Myocardial injury might be due to myocarditis, characterized by infiltrates of interstitial mononuclear inflammatory cells (Xu et al., 2020b) and to a mismatch between oxygen supply and demand [type 2 classification according to the Fourth universal Definition (Thygesen et al., 2018)]. This second option may be secondary to the primary infection, hemodynamic and respiratory derangement.

A clinical and electrophysiological manifestation of myocarditis are arrhythmias, which have been reported in 16,7% of total patients and in 44% of ICU ones (Wang et al., 2020a). Sinus tachycardia is often linked to hypoxemia. The most common arrhythmia seems to be atrial fibrillation (new-onset or permanent with higher rate) which often appears in patients with electrolyte disturbances, ischaemia or acute cor pulmonale (Huang et al., 2020). New onset atrial fibrillation has been associated with higher mortality (Walkey et al., 2014; Boriani et al., 2019). There have been recognized different causes for arrhythmias genesis: first, via cross-talk between immune cells and myocardial cells, resulting in fibrosis that creates slow conduction areas; second, via leukocytes interacting with conduction system cells; third, via antibodies and cytokines causing ionic channels dysregulation. Another CV manifestation is heart failure (HF) which holds the worst presentation and prognosis (Li et al., 2020c; Zhou et al., 2020). It can be due to different mechanisms, such as acute myocardial infarction, myocarditis, acute kidney damage, hypovolemia, dehydration with hypovolemia, Takotsubo cardiomyopathy and ARDS with hypoxemia (Guan et al., 2020). In rare cases, myocarditis may have a fulminant presentation (with cardiac symptoms, haemodynamic deterioration, arrhythmias, elevation of biomarkers and suggesting imaging) (Liu et al., 2020b). HF could evolve in cardiogenic shock.

Laboratory tests in patients at high risk of mortality show high levels of Troponin T, IL-6 (Zhou et al., 2020) and DDimer (Walkey et al., 2014). In addition, the dynamic variations of Troponin I and proBNP have to be considered to identify high risk patients (Liu et al., 2020a). Indeed, persistent elevation and dynamic changes of Troponin I is an independent risk factor of mortality especially in patients with previous cardiovascular diseases (Liu et al., 2020a). BNP and NT-proBNP are usually elevated in patients with severe respiratory distress but they may also express cardiac injury in COVID-19 patients (Christ-Crain et al., 2008). Finally, if D-Dimer level is >1,000 ng/dl it may indicate the presence of pulmonary embolism or disseminated intravascular coagulation in COVID-19 patients (Chen et al., 2020b).

In spite of what has been reported until now, multiple studies have found that the incidence of hospitalization for acute MI has decreased as much as 40–50% during the pandemic (De Filippo et al., 2020; Solomon et al., 2020).

This evenience could have two possible explanations: a patient avoidance of medical care secondary to the fear of being infected if hospitalized and redistribution of health care.

*Treatment* in patients with suspected SARS-CoV-2 infection and acute myocardial infarction, STEMI primary PCI might be postponed up to 60 min than the usual delay (120 min) in order to set all the protective measures; behind this delay, fibrinolysis should be considered. In acute myocardial infarction NSTEMI, cardiac CT should be considered for risk stratification in patients at intermediate and low risk. In patients with chronic coronary syndrome, aspirin should not be stopped because of its anti-inflammatory effect (Rauch, 2020). Statin therapy may be interrupted considering the elevated liver enzymes in some COVID-19 patients and the possible rhabdomyolysis occurring as an adverse event of statins (Xu et al., 2020a).

Hypertension treatment with ACEIs and ARBS is a subject of debate. On one side these drugs could increase the expression of ACE2 receptors, raising the risk of COVID-19 infections (Hamming et al., 2004; Chen et al., 2020f; Hoffmann et al., 2020); on the other side, studies on animal models have shown a protective role of ARBs for lungs affected by some viruses (Rodrigues Prestes et al., 2017). Therefore, right now, there is no evidence of benefit or harm by those drugs, consequently they should not be discontinued or contraindicated (Poissy et al., 2020).

The incidence of pulmonary embolism in COVID-9 patients is reported to be high (Danzi et al., 2020; Poissy et al., 2020) and all COVID-19 patients admitted in hospital should start anticoagulation at prophylactic dose. If clinical and radiological findings confirm pulmonary embolism an appropriate treatment should be started, represented by thrombolysis for patients in shock, anticoagulation with unfractionated heparin, LMWH for stable patients. Regarding NOACs, an interaction with COVID drugs (such as lopinavir/ritonavir via Cytochrome P450) has to be considered, causing an increased bleeding risk. Therefore, it is reasonable to consider to substitute NOAC with LMWH. Vitamin K antagonists should be discontinued and substituted with heparin and only considered in particular conditions such as mechanical valves implant (Guan Yap, 2003).

In COVID-19 patients with arrhythmias management and treatment are influenced by the clinical presentation and considering drug interactions. If allowed by haemodynamic conditions, antiarrhythmic drugs for AF and atrial flutter should be discontinued, due to interactions with azithromycin; therefore, minimal dosage of beta-blockers and calcium channel blockers should be preferred to gain rate control. Otherwise, in case of hemodynamic instability, electrical cardioversion does not seem effective in COVID-19 patients without treating underlying conditions (hypoxaemia, hypokalaemia, hypomagnesaemia, acidosis). When ventricular tachycardia, ventricular fibrillation, AF or atrial flutter occur in unstable patients’ amiodarone can be considered the safest drug, due to his property to not cause QT dispersion. Sotalol and flecainide should not be administered.

QT prolongation, ventricular fibrillation, Torsades de Pointes (TdP) and sudden death are rarely due to a single administration of a drug (Chen et al., 2020e), and even when arrhythmias occur, they often disappear on their own. Hydroxychloroquine - which was mainly used in the early stages of the pandemics - causes significant QT prolongation in association with azithromycin, increasing the incidence of cardiac arrhythmias at 31% (Zhao et al., 2020a). In patients with ventricular tachycardia and QT prolongation electrical, cardioversion and lidocaine represent the treatment of choice especially in patients with antiviral therapy. Drugs inducing QT prolongation should be stopped in patients with QTc >500 ms (550 ms in presence of bundle branch blocks) or an increase >60 ms from the baseline ECG; negative chronotropic drugs (beta-blockers, digoxin, ivabradine and calcium channel blockers), inducing bradycardia, prolong QT interval and their interactions with antiviral drugs need to be monitored.

### A Multiorgan Disease: Involvement of the Nervous System in COVID-19 Infection

Coronaviruses (CoVs) (including also SARS-CoV-2) may invade the central nervous system (CNS) causing neurological diseases. Indeed, in order to gain cell entry, as it has already said, the virus binds to the ACE2 receptor which is also expressed in neurons, vascular endothelial and glial cells (Zhao et al., 2020a).

Two main routes through which the SARS-CoV-2 invades the nervous system have been proposed. Firstly, the dissemination of SARS-CoV-2 in the systemic circulation during an early or later phase can determine cerebral involvement (Baig et al., 2020). Secondly, increasing evidence shows that CoV may first invade peripheral nerve terminals and then gain access to the CNS via a synapse connected route (Li et al., 2012; Li et al., 2020d). Through the trans-synaptic transfer, CoV can access to the brainstem (including the nucleus of the solitary tract and the nucleus ambiguous, which have a fundamental role in control of heart and lung’ function) and this can worsen the dysfunction of the respiratory system (Netland et al., 2008). Nevertheless, this hypothesis has been debated due to the fact that brain failure usually gives a pattern of respiratory failure different from that seen in patients with COVID-19 (Turtle, 2020).

Together with the acute pneumonia and severe respiratory distress symptoms, many patients with COVID-19 complain of neurological disturbances, ranging from headache, hyposmia, ageusia, muscle pain to conscious disturbance, skeletal muscle injury and seizures. Mao et al. (2020) reported that 36% of patients with a severe infection presented various neurologic manifestations involving CNS, PNS and skeletal muscles, mostly in old patients. Some of these neurologic symptoms might be foreseeable. Indeed, it is not uncommon that during an infective disease with high fever patients, especially the older, can manifest seizures. It has also been supposed that the severe hypoxia secondary to acute respiratory distress syndrome can enhance brain damage, being therefore the main reason for CNS involvement (Li et al., 2020d). As far as epilepsy is concerned, clinicians have to be careful in choosing the correct treatment in COVID-19 patients. In fact, they have to consider pharmacological interactions between antiepileptic drugs (AEDs) and COVID-19 drugs (Liverpool Drug Interaction Group, http://www.covid19-druginteractions.org/). The same interactions can underlie seizures occurrence in epileptic patients even if in appropriate treatment. For example, cases reported the association of seizures with chloroquine therapy in systemic lupus erythematosus patients (Krzeminski et al., 2018).

Another neurologic manifestation is delirium. Moreover, as far as COVID-19 is concerned, the use of total-body personal protective equipment by medical staff, artificial light, closed wards, isolation and the absence of relatives can exacerbate and early arise delirium symptoms. Because its presence is associated with a devastating impact in outcomes for critically ill patients it should be promptly recognised and treated, according to current guidelines (Burry et al., 2019). Medical treatment for delirium includes not only supportive medical care and non-pharmacological intervention (which, as said before, in the contest of COVID ward can be difficult), but also antipsychotic drugs (e.g., haloperidol, olanzapine and quetiapine), which need to be used with caution due to the QTc prolongation and their interaction with COVID-19 drugs. Adequate pain identification and management, both in ICU and non-ICU setting, is crucial in order to prevent this manifestation which itself is a robust prognostic indicator of worse survival immediately (Kotfis et al., 2020).

Alongside, cerebrovascular system is also involved, as reported from the description of strokes (both in the setting of critical illness and during hypotension), coagulopathy and antiphospholipid antibodies in patients with COVID-19 (Zhang et al., 2020a) and acute hemorrhagic necrotizing encephalopathy (Poyiadji et al., 2020).

Neurologist should also expect the occurrence of post infectious syndromes such as acute disseminated encephalomyelitis and Guillain-Barré syndrome; the latter has been described even if, actually, it is not known if it is a consequence or a coincidence of SARS-CoV-2 infection, because real-time polymerase-chain-reaction assay of the CSF was negative for SARS-CoV-2 (Toscano et al., 2020; Zhao et al., 2020b). An important fact is the time of onset, which is essential to distinguish acute polyneuropathy with COVID-19 from critical illness neuropathy and myopathy, which usually appear later in the course of intensive care unit recovery.

In addition (and differently from SARS infection), olfactory and taste disorders hold a special interest due to the fact that they have been complained of during the incubation period while sudden onset sensorineural hearing loss has been reported during the course of the Covid-19 (Koumpa et al., 2020; Guan et al., 2020). An Italian cross-sectional survey described that 20% of patients presented olfactory and taste disorders before the hospital admission and only 13% during the hospital stay; interestingly patients with these symptoms were younger than those without (Giacomelli et al., 2020). The exact pathogenesis of ageusia and anosmia is still unknown: it might be due to a direct damage inside the olfactory bulb from the coronavirus or it might express only the classical congestion which is seen also in other viral infections. Interestingly, COVID-19 patients do not report nasal obstruction, differently from flu.

Apart from the suspected neurotropism of SARS-CoV-2, neurologists are concerned about the impact that the infection can have in patients with chronic neurologic diseases (e.g., previous stroke or other neurodegenerative disorder) or in patients with diseases that need immune-modulatory drugs (for example multiple sclerosis, myasthenia gravis, and neuromyelitis optica). In the latter case, if taking off immune-modulatory drugs is not advisable due to the catastrophic complications that this can set off, a possible intervention is to reassess treatment, both in dosage and in frequency of infusion (e.g., natalizumab and fingolimod for multiple sclerosis) (Bomprezzi and Pawate, 2014; Ghezzi, 2019). Time-dependent treatment of acute patients (namely for ischemic stroke) should also be reorganized with the aim to appropriately deal with it and to not increase disability in human beings (Khosravani et al., 2020).

Finally, even if it is known that the most severe neurologic complication occurs later and in more severe patients, with the growing knowledge about SARS-CoV-2 infection, big data, strenuous surveillance and global cooperation in recognizing other acute or post-infectious conditions are needed in order to deal with this challenge in the possible best way.

### A Multiorgan Disease: Psychiatric Implications of COVID-19 Pandemic

Maintaining a satisfactory mental health is a delicate balance that COVID-19 pandemic has undermined for the general population, health care workers, psychiatric patients and patients with COVID-19. During lockdown, the general population have experienced adverse psychological outcomes, such as anger, anxiety, boredom, confusion, fear, depression, emotional exhaustion, frustration, irritability, stress, avoidance behaviour and subthreshold symptoms of alcohol use disorder (Brooks et al., 2020; Pfefferbaum and North, 2020). Excessive concern for the pandemic with distressing somatic symptoms, detachment from others, post-traumatic stress disorder (PTSD), violence and suicidal ideation have also been described (Brooks et al., 2020; Pfefferbaum and North, 2020). Cross-sectional, self-report surveys from January to april 2020 found that these symptoms were clinically significant present in up to 36% of adults (Wang et al., 2020c). Among healthcare workers—who are at high risk of exposure—psychiatric problems, such as significant psychological stress and acute and/or PTSD were more common in workers exposed to the virus than in those who were not (Kisely et al., 2020). In particular, anxiety was present in 12–20%, depression in 15–25%, insomnia in 8% and traumatic distress in 35–49% (Rossi et al., 2020). Among patients with pre-existing psychiatric illness, infection with SARS-CoV-2 may exacerbate the pre-existing illness (Holmes et al., 2020). In addition to respiratory symptoms, COVID-19 patients may present neuropsychiatric syndrome in the acute phase of the illness, such as confusion and impaired consciousness, anxiety (35%) and depression (28%) (Rogers et al., 2020). The pathogenesis of psychiatric symptoms in previous healthy patients may include biologic and psychosocial factors. In fact, it is known that a combination of systemic infection, viral neurotropism and environmental stress facilitates induces development of psychiatric pathologies (Kisely et al., 2020). The “cytokine storm” secondary to viral infection, with high levels of circulating cytokine (IL-6, IL-1β, IL-2, TNF- α), is responsible of symptoms from apathy, motor inhibition to obsessive compulsive disorder, PTSD and schizophrenia (Steardo et al., 2020).

### A Multiorgan Disease: Ocular Involvement of COVID-19 Infection

#### Ocular Findings and Early Diagnosis

According to recent reports, the only ocular clinical manifestation in patients with COVID-19 is acute viral conjunctivitis (Chen et al., 2020d; Wu et al., 2020a; Xia et al., 2020). SARS-CoV-2, as described by Wu et al. (2020a), can cause ocular involvement (32% of 38 COVID-19 patients) and sometimes it may represent the first symptom of COVID-19 disease. The acute nonspecific viral conjunctivitis is characterized by conjunctival hyperemia, chemosis, epiphora, foreign body sensation, tearing and secretions. Chen et al. (2020d) identified in a patient with COVID-19 the signs of the viral conjunctivitis through slit lamp examination: bilateral moderate conjunctival injection, watery discharge, inferior palpebral conjunctival follicles and tender palpable preauricular lymph nodes. Treatment is the same as common viral conjunctivitis. Ocular findings were found in patients with high levels of leukocytes, neutrophils, procalcitonin, CRP and lactate dehydrogenase suggesting a correlation between ocular involvement and a severe disease form (Wu et al., 2020a).

Moreover, in patients with positive nasopharyngeal swabs for SARS-CoV-2 conjunctival swab was performed resulting positive only in a small part of patients (5%) with conjunctivitis (Wu et al., 2020a). Additionally, Vinores et al. (2001) evaluated the tear and conjunctival secretions of COVID-19 patients with RT-PCR and only one swab on 30 tested positive for SARSCoV- 2.

Based on these results, SARS-CoV-2 can cause ocular complications and in some cases may represent the first symptom of disease, even if is not a common manifestation. Early screening of SARS-CoV-2 in patients with conjunctivitis by searching the virus in the tears and conjunctival secretions may be conceivable. However, since the viral RNA levels in conjunctival specimens are dramatically lower than those in respiratory samples (Chen et al., 2020d), the conjunctiva might not serve as an ideal site for early diagnostic tests of SARS-CoV-2 infection.

Regarding other ocular complications, since coagulation disorders are also common in patients with SARS-CoV-2 infection, recent studies have linked coronavirus infection with retinal disorders, such as microangiopathy (Invernizzi et al., 2020b), hemolytic uremic syndrome with retinal vessel occlusion (Greenwood, 2015) and impending central retinal vein occlusion (Invernizzi et al., 2020a). Marinho et al. (2020) reported an alteration of inner retinal layers, such as hyperreflective lesions, based on optical coherence tomography (OCT) scans. However some authors suggested a possible misinterpretation of these findings, which may represent an individual variability of normal retinal vessels (Vavvas et al., 2020). The “Screening the retina in patients with COVID-19” study (SERPICO-19) showed the presence of retinal findings in patients with COVID-19, including retinal haemorrhages (9.25%), cotton wools spots (7.4%), drusen (11.1%), dilated veins (27.7%) and tortuous vessels (12.9%) (Invernizzi et al., 2020b). However, concerns may be raised about the presence of bias in the sample enrolled, given the high prevalence of hypertension and diabetes in the cohort, which make these findings as possible incidental findings.

Further clinical studies are needed to evaluate the clinical spectrum of ocular diseases caused by SARS-CoV-2. Moreover, since ACE-2 is a cellular receptor for SARS-CoV-2 (Lu et al., 2020b) detected in the human retina (Wagner et al., 1996; Senanayake et al., 2007), a possible involvement of the internal ocular structures such as the retina cannot be excluded.

#### Transmission Through the Ocular Surface

The role of the eye in transmitting human SARS-CoV-2 is still under discussion.

Some authors have underlined that the transmission through the ocular surface should not be underestimated, since infectious droplets can easily contaminate the human conjunctival epithelium (Lu et al., 2020a). The detection of the SARS-CoV-2 in tears and conjunctival secretions confirms this hypothesis (Chen et al., 2020d; Wu et al., 2020a; Xia et al., 2020). However, the low prevalence of SARS-CoV-2 in the ocular surface of patients with conjunctivitis and the absence in patients without ocular signs could mean that tears and conjunctival secretions of COVID-19 patients are not a common infectious route for SARS-CoV-2. Nevertheless, the risk of transmission could not be completely eliminated. As reported by Chen et al. (Chen et al., 2020d) the viral loads in conjunctival specimens of COVID-19 patients gradually decrease over time with less potential for transmissibility accompanied by improvement of the ocular symptoms. Therefore SARS-CoV-2 in conjunctival specimens may represent a source of spread, especially in the acute stage of ocular complications characterized by high viral load. Qing et al. (2020) stressed the role of lacrimal drainage as a route of SARS-CoV-2 transmission. Anatomically, the ocular surface and upper respiratory tract are connected by nasolacrimal duct. Therefore, it is possible that the virus reaches the tears through droplets, passing through the nasolacrimal ducts and then into the respiratory tract.

#### Precautionary Measures Needed for Physicians

Containing viral spread is the primary means by which we protect people from newly emerging infections (Sommer, 2020). Ophthalmologists are a high-risk category, not only because they have close contact with patients during the examination (conjunctival, tear secretions and aerosol secretions), but also because their daily outpatient clinic and emergency lists have a high patient volume (Lai et al., 2020a; Romano et al., 2020). In order to minimize transmission of COVID-19, some precautionary measures are mandatory for physicians when coming into contact with suspected or confirmed cases of COVID-19 (Lai et al., 2020a; Lai et al., 2020b; Li et al., 2020a; Mungmungpuntipantip and Wiwanitkit, 2020; Romano et al., 2020). These measures include:

- Protection of health workers with appropriate PPE: protective eyewear can prevent direct inoculation of respiratory droplets through the conjunctiva, and also indirect contamination of conjunctiva through inadvertent eye rubbing with a contaminated hand. During eye examination, a self-made transparent polycarbonate protector mounted to the slit lamp offers a physical barrier between the patient and physician (Wan et al., 2020b). Non-contact air-puff tonometry has been associated with a micro-aerosol formation (Wan et al., 2020b); therefore, other ways of intraocular pressure measurement, such as i-Care tonometry or Goldmann applanation tonometry should be used instead
- Appropriate environmental control: important to reduce the concentration of virus on contaminated surfaces. Considering that coronavirus can persist on inanimate surfaces up to 9 days (Kampf et al., 2020), it is crucial to perform an appropriate sanitation of the potentially contaminated environment. Equipment must be cleaned and disinfected after every clinic session.
- Reorganization of the workflow to minimize the risk of cross infections: non-urgent consultations and operations should be delayed. Urgent consultations (ocular trauma, acute glaucoma, retinal detachment, alkali chemical injury, etc.) should be attended with adequate PPE.

#### Management of COVID-19 Patients in the Intensive Care Unit

Hospitalization in ICU is required in about 5% of COVID-19 patients who can rapidly progress to ARDS, MOF, sepsis and septic shock. The primary reason for ICU admission is the patient’s need for endotracheal intubation and mechanical ventilation (Grasselli et al., 2020).

COVID-19 patients mainly are affected by respiratory system failure whereas other organ functions are less involved. The most frequent clinical evolution during the hyperinflammatory phase is the development of ARDS. Nevertheless, not all the cases of severe ARF are considered as typical ARDS. For this reason, Marini et al., called ARDS COVID related as C-ARDS. There are differences between COVID-19-related ARDS and ARDS caused by other factors as defined by Berlin criteria, and, therefore, there are also differences in the treatment (Li and Ma, 2020).

ARDS can be classified on Berlin criteria in (The ARDS Definition Task Force, 2012):

- *Mild ARDS:* 200 mmHg < PaO2/FiO2 ≤300 mmHg (with PEEP or CPAP ≥5 cmH2O, orunventilated).
- *Moderate ARDS:* 100 mmHg < PaO2/FiO2 ≤200 mmHg (with PEEP ≥5 cmH2O, or unventilated).
- *Severe ARDS:* PaO2/FiO2 ≤100 mmHg (with PEEP ≥5 cmH2O, or unventilated). When PaO2 is not available, SpO2/FiO2 ≤315 suggests ARDS (including unventilated patients) (Circolare Ministeriale, 2020; World Health Organization, 2020a; European Center for Disease Prevention and Control, 2020; Ranieri, 2012; Arabi, 2020; World Health Organization, 2020b; Wax, 2020; BPC-PDTA, 2020).

#### Invasive Mechanical Ventilation

Patient selection for invasive mechanical ventilation (IVM) is clinically based on severe hypoxemia and dyspnea in patients previously treated by non invasive ventilation (NIV) or continuous positive airway pressure (CPAP) and, most of the time, the timing of IVM is very important.

Most of the patients in intensive care units shows the same clinical findings of acute respiratory distress syndrome but in some cases they do not have the same response to protective ventilation.

Mechanical ventilation in COVID-19 patients results in different respiratory patterns which can be challenging.

In april 2020, Marini and Gattinoni (2020) laid out a conceptual model to underline the role of a possible endothelial damage that disrupts pulmonary vasoregulation leading to a ventilation-perfusion mismatch and thrombogenesis. The endothelial damage could clinically translate into a particular pattern characterized by hypoxemia with normal pulmonary compliance, findings uncommon for ARDS patients (Gattinoni et al., 2020a).

This discrepancy between pulmonary compliance and hypoxemia may lead to different ventilation settings based on the interactions between different factors: the phase of infection, the host response, and the time of NIV/CPAP.

The result of this interaction lead to a time-related disease spectrum within two primary “phenotypes” named (Gattinoni et al., 2020b): Type “L” patients, with Low elastance, Low ventilation to perfusion ratio, low lung weight, and low recruitability; IVM in this type of patients is aimed to minimize pulmonary stress, reduce hypoxemia and interrupt the vicious cycle that may lead to a ventilator-induced lung injury (VILI) (Marini and Gattinoni, 2020); Once intubated and sedated, these patients, present a good tolerance to Tidal Volume (TV) 7–8 ml/kg and they are low responsive to PEEP; a worsening of clinical symptoms and signs might be related with the negative intrathoracic pressure associated and the increased tidal volume in spontaneous breathing (Gattinoni et al., 2020a). Also, prone positioning should be used only as a rescue maneuver. Type “L” patients can evolve towards the phenotype “H”. Type “H” patients are characterized by High elastance, High right-to-left shunt, High lung weight, and High recruitability. Type H patients should be treated as severe ARDS, including protective lung ventilation setting and higher PEEP, prone positioning and extracorporeal support (Brochard et al., 2017). The aim of mechanical ventilation in Type H patients is to minimize lung stress and ventilation-perfusion mismatch (Marini and Gattinoni, 2020).

Type L and Type H patients are best identified by CT scan and are affected by different pathophysiological mechanisms. Considering these assumptions, invasive ventilatory approach should be evaluated and above all, differentiated both in acute respiratory failure and in post acute phase. A clinical protocol should be applied in each COVID-19 center in order to differentiate patients that need invasive ventilation treatment and, above all, to choose which patients would benefit from invasive ventilation in relation to the stage of the disease and patient phenotype (Marini and Gattinoni, 2020).

#### Hemodynamic Support in Septic Shock

In septic COVID-19 critical patients, the illness is characterized by an organ dysfunction caused by a dysregulated response of the host to suspected or certain infection, with Sequential [Sepsisrelated] Organ Failure Assessment (SOFA) score of two points or more (Singer et al., 2016). The signs of organ dysfunction include altered mental status, difficult or rapid and superficial breathing, low oxygen saturation, oligoanuria, tachycardia, weak pulsations, cold extremities or hypotension, skin alterations, laboratory findings of coagulation alterations, thrombocytopenia, acidosis, elevated lactates or hyperbilirubinemia.

These COVID-19 critical patients may evolve to septic shock, defined as hypotension unresponsive to volume expansion, which requires vasopressors to maintain MAP ≥65 mmHg and serum lactate level ≥2 mmol.

The frequency of septic shock varies from 20 to 35% in ICU among patients affected by COVID-19 (Wang et al., 2020a; Yang et al., 2020). In some studies, the development of fulminant myocarditis has been possibly the dominant reason for 40% of ICU deaths (Ruan et al., 2020). Other studies also advise that risk factors to consider are older age comorbidities like diabetes and cardiovascular diseases including hypertension, lower lymphocyte count, higher D-dimer level, or possible cardiac injuries (Wang et al., 2020a; Yang et al., 2020).

The two mainstays of hemodynamic treatment have been increasing intravascular volume with fluids and by counteracting hypotension, as well as low cardiac output with vasoactive drugs with varying inotropic properties. The use of dynamic assessment should guide fluid therapy and it may reduce mortality, duration of mechanical ventilation and ICU length of stay (LOS). Within their respective limitations, the functional hemodynamic parameters which should be used to guide fluid therapy as part of goal directed therapy strategies are parameters such as stroke volume variation (SVV), pulse pressure variation (PPV). In contrast, assessing fluid responsiveness with passive leg raising manoeuvre, central venous pressure (CVP), and mean arterial pressure (MAP) may result in false-negative cases.

Moreover, early lactate clearance-directed therapy (even though a high lactate level does not always imply hypovolemia) may be linked to a reduction in mortality and LOS in ICU, when compared to the central venous oxygen saturation (ScVO2) guided therapy (Pan et al., 2019).

Fluid therapy used to correct circulatory failure is elementary and cheap. However, there are no indications that the fluids should be carefully prescribed in order to maximize their result or limit their side-effects. The use of dynamic assessment to guide fluid therapy has reduced both mortality and duration of mechanical ventilation (Bentzer et al., 2016; Bednarczyk et al., 2017). Although a review that compared restricted to liberal fluid volumes in the initial resuscitation of patients with sepsis has not found any statistically significant variation in mortality or serious adverse events (Meyhoff et al., 2020), we recommended an initial conservative approach to fluid resuscitation in COVID-19 patients with shock. There is no outcome that preferred the use of colloids when compared to the use of crystalloids in critically ill patients (Lewis et al., 2018). Knowing that some colloids are harmful, they are more expensive, and their availability can be limited. Therefore, we recommend the use of crystalloids for fluid resuscitation in COVID-19 patients with shock, using buffered/balanced crystalloids over the unbalanced ones, instead of choosing hydroxyethyl starches, gelatines, or dextrans. Also, the regular use of albumin for initial resuscitation is not linked to improved outcomes (Lewis et al., 2018).

The best first-line treatment on COVID-19 patients with shock is norepinephrine, alternatively, vasopressin or epinephrine should be considered (Gamper et al., 2016; Moller et al., 2016). Dopamine should be avoided, as it increases the arrhythmias risks. The targeted therapy based on the standard of care MAP targeted of 60–65 mmHg, titrating the vasoactive agents is recommended (Moller et al., 2018); moreover, it is also suggested to add a second-line agent (vasopressin) if the target is not achieved by norepinephrine itself (Honarmand et al., 2020). Furthermore, based on a physiological reason, the use of dobutamine in COVID-19 patients with shock and cardiac dysfunction, should be considered (Moller et al., 2018).

If available, Guideline recommends that all patients who require vasopressors have an arterial catheter placed as soon as practical.

For adults with COVID-19 and refractory shock, it is recommended the use of low-dose corticosteroid therapy (“shock-reversal”) over no corticosteroid. A typical corticosteroid regimen in septic shock is intravenous hydrocortisone 200 mg per day administered either as an infusion or intermittent doses. The duration of hydrocortisone therapy is usually a clinical decision (https://www.covid19treatmentguidelines.nih.gov/critical-care/hemodynamics/; Rhodes et al., 2016; Bednarczyk et al., 2017).

#### Patient’s Step-Down From the Intensive Care Unit

COVID-19 patients may be stepped-down from ICU to medical wards (or ad-hoc COVID-19 wards) when they show a non-critical condition and an improvement of clinical features and radiologic findings. The aims of the non-ICU department include the weaning from oxygen or from the use of CPAP/NIV or helmet CPAP, the prosecution of treatment of bacterial superinfection eventually contracted in ICU, the prevention of possible complication of Sars-CoV-2 infection, the follow-up of patients until hospital discharge. Whenever possible, patients should be discharged from hospital after recovery confirmed by the double consecutive negative swabs (Procedura Regionale Nuovo Coronavirus Sars, 2020).

Most patients admitted to ICU have a prolonged length of stay (on average 3 weeks), therefore requiring adequate rehabilitation once stepped-down to medical wards (Procedura Regionale Nuovo Coronavirus Sars, 2020). Despite the progressive clinical improvement of the respiratory disease, prolonged bed rest syndrome and invasive mechanical ventilation sequelae (such as iatrogenic post-intubation dysphagia, tracheostomy management) have been reported. Hence, it is advisable to promote a rehabilitation program into the non-ICU department (aerobic exercise, strength training for muscle weakness, bronchial clearance techniques in hyper-secretive patients) and to direct the frailest patients with severe sequelae to rehabilitation units (Brugliera et al., 2020).

#### Hospital Discharge

Different rules have been developed to decide whether or not patients should be discharged home after hospitalization. Generally, independently from the ICU or non-ICU stay, two main strategies are indicated (CDC Discontinuation, 2020):

Test-based strategy:

Resolution of fever without the use of fever-reducing medications and improvement in respiratory symptoms (e.g., cough, shortness of breath), and negative results of a COVID-19 molecular assay for detection of SARS-CoV-2 RNA from at least two consecutive nasopharyngeal swab specimens collected ≥24 h apart (total of two negative specimens).

Non-test-based strategy:

At least 3 days (72 h) have passed since recovery defined as resolution of fever without the use of fever-reducing medications and improvement in respiratory symptoms (e.g., cough, shortness of breath); and at least 7 days have passed since symptoms first appeared.

It is therefore specified, in accordance with what is outlined by the CDC, that meeting criteria for discontinuation of transmission-based precautions is not a prerequisite for discharge.

In Italy, a COVID-19 patient is considered cured after the resolution of symptoms and two negative tests for SARS-CoV-2 at 24-h intervals. In patients who clinically recover before 7 days after onset, an interval of 7 days between the first and the final test is recommended. For virus clearance it is defined as a negative viral RNA from body fluids of symptomatic and asymptomatic patients, accompanied by the appearance of specific IgG (Ministero della salute, 2020).

As a precautionary measure, in several countries patients are told to self-isolate once discharged from the hospital, even in case of swab negativity (Ministero della salute, 2020). Serological testing performed at time of discharge can provide important information on the immune response of infected individuals (Ministero della salute, 2020).

### Nursing Role During COVID-19

In the setting of hospital care, all healthcare workers, including nurses, technicians, and drivers have played an important and variegated role during pandemic months. In regard to nurses, they helped doctors not only in treating COVID-19 patients, but also in supplying nosocomial infection prevention and surveillance (Chen et al., 2020c). Moreover, they provided health and screening education and support for the general population and high-risk categories (Chen et al., 2020c).

## Conclusions

The COVID-19 pandemic has challenged healthcare systems on a global scale, requiring that hospitals make a significant effort to repurpose their services and healthcare delivery. As the pandemic has progressed, clinicians have developed a greater understanding of the multifaceted nature of COVID-19 disease, as well as its myriad presentations not limited to the respiratory tract. Given the complex nature of this new condition, assessment and treatment of hospitalized patients should involve the expertise of a range of specialties. Knowledge-sharing between specialists is undoubtedly required to determine the timing and setting in which proven treatments should be administered to manage patients suffering from COVID-19.

## Author Contributions

All authors contributed equally to the review of literature and to the production of this manuscript.

## Conflict of Interest

The authors declare that the research was conducted in the absence of any commercial or financial relationships that could be construed as a potential conflict of interest.
